# Bioactivity-Guided Isolation of Neuritogenic Factor from the Seeds of the Gac Plant (*Momordica cochinchinensis*)

**DOI:** 10.1155/2018/8953958

**Published:** 2018-05-31

**Authors:** E. Mazzio, R. Badisa, S. Eyunni, S. Ablordeppey, B. George, K. F. A. Soliman

**Affiliations:** College of Pharmacy & Pharmaceutical Sciences, Florida A&M University, Tallahassee, FL 32307, USA

## Abstract

Nerve growth factor (NGF) is an endogenously produced protein with the capacity to induce central nervous system (CNS) neuronal differentiation and repair. NGF signaling involves its binding to tropomyosin-related kinase (Trk) receptors, internalization, and initiation of phosphorylation cascades which cause microtubule reorganization and neuronal outgrowth. Because NGF cannot cross the blood-brain barrier, its therapeutic use is limited. Synthetic peptides that can act as NGF receptor agonists (NGF mimetics) are known to attenuate neurodegenerative pathologies in experimental models of Alzheimer's disease and Parkinson's disease; however, the existence of plant-based NGF mimetics is uncertain. For this reason, we recently completed a high throughput screening of over 1100 nutraceuticals (vitamins, herbal plant parts, polyphenolics, teas, fruits, and vegetables) to identify neuritogenic factor using a PC-12 cell model. Remarkably we found only one, commonly known as the seed of Gac plant (*Momordica cochinchinensis)* (MCS). In the current study, we further investigated this seed for its neuritogenic effect using bioactivity-guided chemical separations. The data show no biological neuritogenic activity in any chemical solvent fraction, where activity was exclusive to the crude protein. MSC crude proteins were then separated by 1D electrophoresis, where the active neuritogenic activity was confirmed to have a molecular mass of approximately 17 kDa. Subsequently, the 17kDa band was excised, digested, and run on a UPLC-MS/MS with a Q Exactive Hybrid Quadrupole-Orbitrap Mass Spectrometer with data evaluated diverse tools such as X! Tandem, OMS, and K-score algorithms. Proteomic evaluation of the 17kDa band confirmed evidence for 11S globulin subunit beta, napin, oleosin, Momordica trypsin inhibitors (TI) MCoTI-I /II, and many isoforms of Two Inhibitor Peptide Topologies (TIPTOPs). While all peptides identified correspond to the genus/species,* Momordica cochinchinensis* and* Cucumis Sativus*, a significant limitation of the analysis is the nonexistence of full annotation for the* Momordica cochinchinensis* proteome. In conclusion, these findings demonstrate that there is a stable protein within MCS having a mass of 17kDa with the capacity to induce neurite outgrowth. Future work will be required to establish the therapeutic value of the MCS for the treatment of neurodegenerative diseases.

## 1. Background

Endogenously produced neurotrophins are continuously being discovered such as nerve growth factor (NGF), brain-derived neurotrophic factor (BDNF), ciliary neurotrophic factor (CNTF), glial cell line-derived neurotrophic factor (GDNF), cerebral dopamine neurotrophic factor (CDNF), mesencephalic astrocyte-derived neurotrophic factor (MANF), and neurotrophins 3/4. All of these neurotrophins evoke central nervous system (CNS) neuron differentiation, growth, axon regeneration, and repair [1–7]. Many of these are peptides which are of large mass and impermeable through the blood-brain barrier (BBB) [8] and can induce severe side effects such as peripheral neuropathies. As such, the limited use of full-length active neurotrophins is restricted to primary applications in genetically modified stem cell transplants [4, 9, 10], artificial biomaterial nerve guidance systems [11, 12] targeted delivery nanoparticle drug systems [13], or administration through recombinant viral vectors [14].

Neurotrophic factor mimetics would be attractive drug candidates due to their ability to augment neuronal survival and attenuate age-related degenerative conditions, but very few are in existence. Of the few, are synthetic dimeric dipeptide mimetics (loop 4 of NGF) which augment neuronal survival in models of Alzheimer's disease, Parkinson's disease [15, 16], hemorrhagic stroke, and global cerebral ischemia [17, 18]. For this reason, we recently completed a high throughput screening of over 1100 nutraceuticals (polyphenolics, crude herbs, vitamins, seeds, nuts, fruits, vegetables, etc.) to determine if there any plant or traditionally used natural medicines that contain a neuritogenic factor [19]. The results were remarkable in that we only found a single hit, which was the crude extract of Gac fruit seed (aril removed), with a botanical name of* Momordica cochinchinensis (MCS)*. While the seeds (MCS) have been used in traditional Chinese medicine to treat arthritis, muscle cramps, and hemorrhoids, very little information exists on seed component to provide disease-modifying effects in any major age-related neurodegenerative model. In this study, we continue to explore the nature of the unknown neuritogenic factor within the MCS responsible for driving neurite outgrowth in PC-12 cells.

## 2. Methods and Materials

Hanks Balanced Salt Solution, (4-(2-hydroxyethyl)-1-piperazine ethane sulfonic acid) (HEPES), ethanol, 96 well plates, rat tail collagen, collagen-coated plates, nerve growth factor, general reagents, and supplies were all purchased from Sigma-Aldrich Co. (St. Louis, MO, USA) and VWR International (Radnor, PA, USA). The* gac (*Momordica cochinchinensis) seeds were purchased from Plum Flower Brands and Mayway Traditional Chinese Herbs (Oakland, California, USA).

### 2.1. Cell Culture

PC-12 cells were obtained from ATCC (Manassas, VA). The cells were cultured at 37°C in 5% CO_2_/atmosphere and grown in RPMI-1640 with 10% heat-inactivated horse serum, 5% fetal bovine serum, and penicillin/streptomycin (100 U/ 0.1 mg/ml). For experiments, cells were disbursed into a homogenous solution of cells and plated at a density of approximately 0.1×10^5^ cells/ml on 96-well collagen-coated plates and incubated for seven days.

### 2.2. MCS Seed Extractions

#### 2.2.1. Solvent Extractions: Method 1

Chemical extractions of MCS were carried out using absolute ethanol, ether, and ethyl acetate. Solvents were evaporated, the residual was reconstituted in ethanol, and dilutions were prepared in sterile HBSS for cell culture. The working concentrations were 0.0020, 0.004, 0.01, 0.02, 0.03, 0.06, 0.13, 0.26, 0.52, and 1.04 mg starting crude seed /ml. All samples were compared to NGF and crude seed extract at 0.2 mg/ml for capacity to drive neurite outgrowth.

#### 2.2.2. Protein Extraction: Method 2

The plant total Protein Extraction Kit PE0230 (Sigma-Aldrich, St. Louis, MO, USA) was used to isolate crude seed protein. We saved all washes in addition to the final protein fraction collected for* in vitro* neuritogenic testing. Briefly, 350 mg of MCS was homogenized in 1 ml of ethanol, samples were centrifuged, and ethanol was removed. The seed residue was then washed (vortexed 15-30 seconds) 3x with 1 ml of methanol and centrifuged at 16,000 x g for 5 minutes at 4°C. Supernatant wash solutions were collected, and the pellet was further extracted. Given the dark green color of the methanol extract, the pellet was further washed 3x with 20mls of methanol as a modification to the protocol, to remove all visually evident seed color chemicals. This protocol was followed by a final 2x wash in 1.5 ml of acetone. The washouts contained polyphenolics, tannins, and other plant chemical substances. The remaining seed residue was dried, weighed and then resuspended in the chaotropic solubilizing protein isolating reagent with a 4ul reagent for each mg seed, and vortexed intermittently for 15 minutes. The sample was centrifuged at 16,000 x g for 30 minutes at 4°C, and supernatant (total protein) removed. All collected solvent extracts from the above procedure were evaporated and redissolved in 1 ml of absolute ethanol, diluted in HBSS where working concentrations for biological testing were v/v 0.0020%, 0.004%, 0.01%, 0.02%, 0.03%, 0.06%, 0.13%, 0.25%, 0.50%, and 1.00%. The remaining protein isolated fraction was also tested by dissolving in sterile HBSS 1:5, diluting over a thousandfold range. Working with an unknown, these studies were designed to cover all extracts over many serial dilutions to find the active fraction. These studies indicated that the active component was* not* chemical and exclusive to only the crude protein isolate.

### 2.3. Protein Separation and Electroelution

Both the crude isolated protein (native) and reduced (denatured) were separated by electrophoresis for purification and isolation. For denatured samples, seed protein isolate was combined with a Laemmli sample buffer containing 2-mercaptoethanol, (1:1) then boiled for 5 minutes @ 100°C. Both native and denatured proteins were loaded on to the gel (30ul), then separated using either a 4–20% or an 8-16% Mini-PROTEAN® TGX™ gel: with voltage settings at 200V for 45 minutes, using a standard western blot SDS tris-glycine running buffer. Gels were stained with Blue-bandit ®, washed with deionized water, and bands were excised from the entire gel.

Each gel band slice was placed in 20% ethanol in HBSS and electro-eluted back into solution within siliconized microcentrifuged tubes at 200V. The timing of electroelution varied with molecular weight but was sustained until each blue band was eluted from the gel into solution. These samples were then reconstituted in HBSS and evaluated for biological activity in PC-12 cells. Subsequently, any potential hit (defined as any observation of neurite spindle shape or neurite outgrowth however minor) was resectioned/ reeluted and reevaluated for neuritogenic effects in a dose-dependent fashion. All gel sections by process of procedural elimination left two tiny biologically active (nonvisual) bands.

#### 2.3.1. Proteomic UPLC-MS/MS Analysis

The 15-17kDa gels spots were further digested and evaluated by UPLC-MS/MS–using a Q Exactive™ Hybrid Quadrupole-Orbitrap Mass Spec conducted by Bioproximity LLC (Chantilly, VA, USA). UPLC: Thermo Easy-nLC 1000 Column:C18 reversed phase 50 cm (length) by 75 microns (inner diameter) with integrated nanoelectrospray tip, heated to 50 C Gradient: determined by assay (20 min - 4 hours) at 300 nL/ min, Source: Thermo Easy SprayMS/MS: Thermo Q Exactive quadrupole-Orbitrap mass spectrometer. Data is searched by up to three tandem mass spectrometry protein identification algorithms, including X! Tandem, OMSSA / K-score, and X! Hunter. Further analysis of each sequence was conducted with a Basic Local Alignment Search Tool (BLAST).

#### 2.3.2. In-Gel Digestion

15-17 kDa gels spots were cut into 1 mm_3_ pieces and washed twice with MilliQ water. The gel was destained using 1:1 methanol:50 mM ammonium bicarbonate for 1 min, twice. The gel pieces were dehydrated for 5 min using 1:1 acetonitrile: 50 mM ammonium bicarbonate followed by acetonitrile for the 30s. The gel pieces were dried in a Speed Vac for 10 min. The gel pieces were rehydrated in 5 mM dithiothreitol, 50 mM ammonium bicarbonate and incubated at 56 °C for 20 min. After discarding the supernatant, the gel pieces were incubated in 15 mM iodoacetamide at RT for 20 min in the dark. The gel pieces were washed 2x with water and dehydrated and dried as before. The dried gel pieces were rehydrated in 50 mM ammonium bicarbonate containing 250 ng of mass spectrometry-grade trypsin or chymotrypsin (Promega) and incubated overnight at 37 C. Following digestion; the reaction mixture was acidified to 1% trifluoroacetic acid and desalted.

#### 2.3.3. Peptide Desalting

The digested peptides were desalted using C18 stop-and-go extraction (STAGE) Tips. Briefly, for each sample, a C18 STAGE Tips was activated with methanol, then conditioned with 60% acetonitrile, 0.5% acetic acid followed by 2% acetonitrile, 0.5% acetic acid. Samples were loaded onto the tips and desalted with 0.5% acetic acid. Peptides were eluted with 60% acetonitrile, 0.5% acetic acid and lyophilized in a Speed Vac (Thermo Savant) to near dryness, approximately two Hr.

#### 2.3.4. Liquid Chromatography-Tandem Mass Spectrometry

Using UHPLC-MS/MS (Easy-nLC 1000 UHPLC system, Thermo) was used for each digestion analysis. Mobile phase A was 97.5% MilliQ water, 2% acetonitrile, 0.5% acetic acid. Mobile phase B was 99.5% acetonitrile, 0.5% acetic acid. The 20 min LC gradient ran from 0% B to 35% B over 10 min, then to 80% B for the remaining 10 min. Samples were loaded directly to the column. The column was 15 cm x 75 um ID and packed with 2-micron C18 media (Thermo Easy Spray PepMap). The LC was interfaced to a quadrupole-Orbitrap mass spectrometer (Q Exactive, Thermo Fisher) via nanoelectrospray ionization using a source with an integrated column heater (Thermo Easy Spray source). The column was heated to 50 C. An electrospray voltage of 2.2 kV was applied. The mass spectrometer was programmed to acquire, by data-dependent acquisition, tandem mass spectra from the top 20 ions in the full scan from 400 - 1200 m/z. Dynamic exclusion was set to 15s, singly charged ions were excluded, isolation width was set to 1.6 Da, full MS resolution to 70,000 and MS/MS resolution to 17,500. The normalized collision energy was set to 25, automatic gain control to 2e5; max fills MS to 20 ms, max fills MS/MS to 60 ms and the underfill ratio to 0.1%.

#### 2.3.5. Data Processing and Library Searching

Mass spectrometer raw data files were converted to MGF format using MS convert. Detailed search parameters were printed in the search output XML files. Briefly, all searches required 10 ppm precursor mass tolerance, 0.02 Da fragment mass tolerance, strict tryptic cleavage, 0 or 1 missed cleavages, fixed modification of cysteine alkylation, variable modification of methionine oxidation and expectation value scores of 0.01 or lower. MGF files were searched using the specified sequence libraries. MGF files were examined using X! Tandem using both the native and K-score scoring algorithms and by OMSSA. All searches were performed on Amazon Web Services-based cluster compute instances using the Proteome Cluster interface. XML output files were parsed, and nonredundant protein sets determined using Proteome Cluster. Mass spectrometer RAW data files were also converted to mzXML format using MS convert, processed through several databased using Peaks 8 Studio Peaks 8 Suite (Bio-Informatics Solutions Inc., ON, Canada).

### 2.4. Immunocytochemistry and Fluorescence Microscopy

PC-12 cells were fixed in 4% paraformaldehyde for 15 minutes and subsequently permeabilized in 0.25% Triton X -100 prepared in phosphate buffered saline (PBS) for 15 minutes. Photographic images reflect neurite outgrowth visualized using Molecular Probes® Neurite Outgrowth Staining Kit (Life Technologies, Thermo Fisher Scientific, Norcross GA, USA). Cytoskeletal changes were captured using live morphological imaging, and neurofilament 200kD was determined using immunocytochemistry in fixed, permeabilized cells, with primary rabbit anti-rat, conjugated to goat anti-rabbit Alexa Fluor® 488 with nuclear counterstain of propidium iodide. Samples were analyzed photographically using a fluorescent /inverted microscope, CCD camera, and data acquisition using ToupTek View (ToupTek Photonics Co., Zhejiang, China).

## 3. Results

**Figure 1:**
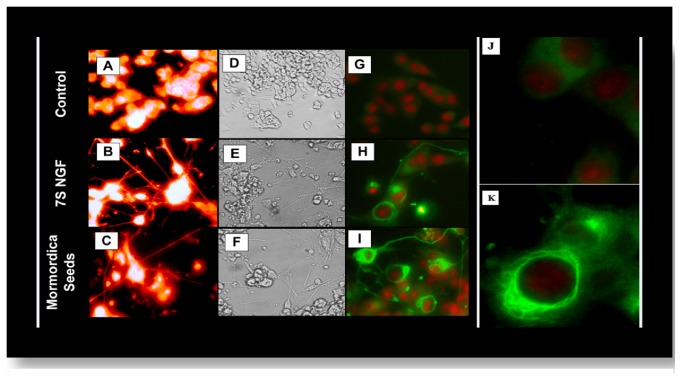
Effects of crude MCS: neurite outgrowth of PC-12 cells at 7 days grown on collagen-coated plates: controls (top), 7S NGF 0.5 *µ*g/mL (mid), and MCS extract (150*µ*g/mL) (bottom). Fluorescent neurite outgrowth imaging using Molecular Probes® Neurite Outgrowth Staining Kit** (A, B, C).** Morphology** (D, E, F)** and changes in neurofilament NF-200 kDa obtained by ICC: primary rabbit anti-rat NF-200 kDa, secondary goat anti-rabbit Alexa 488, and nuclear counterstained with propidium iodide in fixed permeabilized cells (G, H, I) with magnified images (J) control (K) MCS seed.

Gac seeds were pulverized, homogenized, dissolved in ethanol (50mg/ml), then diluted in HBSS, and validated for neuritogenic effects in PC-12 cells (150ug/ml). The impact of* whole crude* MCS aqueous extract (including the residue) is very similar to 7S NGF concerning neurite outgrowth. The results are shown in**Figure 1** relative to controls and NGF treated cells**. In Figure 1**A–C show basic morphology, D–F show neurite extensions, and G–I show the dimensional alterations/expression of neurofilament 160/200 kD (G–I)/J-K (magnified image), which are concentrated around the plasma membrane, branching outward into neurite shafts. These changes were reported to be associated with regeneration, late differentiation, and synaptic plasticity of various neurons [20, 21].

**Figure 2:**
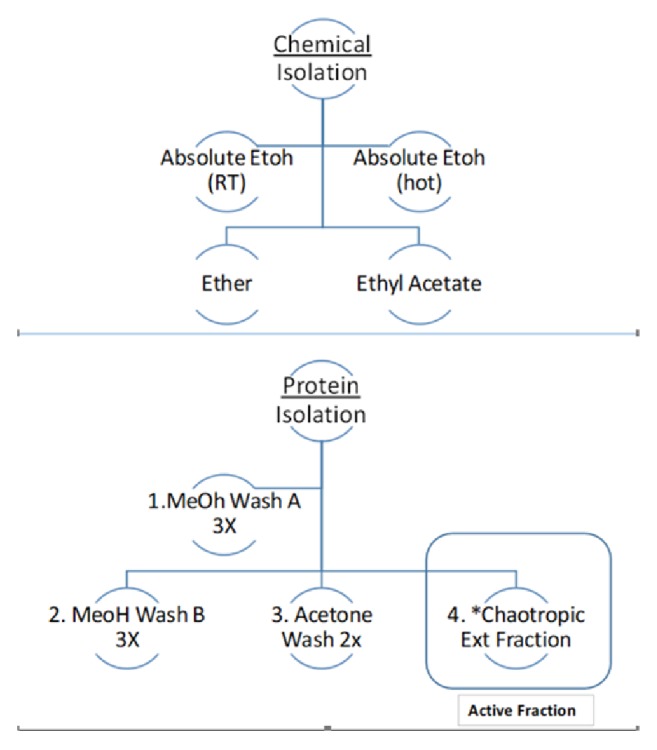
Separations: extraction method 1 (chemical): chemical extractions of MCS seeds were carried out using absolute ethanol, ether, hot ethanol, and ethyl acetate. Solvents were evaporated, reconstituted in ethanol and dilutions prepared in HBSS. Fractionation Schematic Solvent/Protein Extraction Method 2 (protein): Plant Total Protein Extraction Kit PE0230 (Sigma-Aldrich, St. Louis, MO) was used for isolation, and all washes were kept for analysis. All washings as well as the protein isolate were evaporated and diluted in HBSS and evaluated for neuritogenic activity in PC-12 cells. All chemical fractions, methanol, and acetone washes failed to produce neuritogenic effects.

To identify the constituent within the crude seed responsible for neurite outgrowth, the seeds were then subjected to a series of chemical extractions using absolute ethanol, ether, and ethyl acetate** (Figure 2**). After crude extraction, solvents were evaporated and reconstituted in ethanol and diluted in HBSS over a dose range of .002–1.04 mg/ml, then tested for neurotrophic properties* in vitro.* While there was no neuritogenic activity in any chemical fraction, the crude protein isolate from the seed was active (Figure 2: bottom panel: Method 2). Furthermore, we found no mimetic activity in any chemical wash step during the protein isolation procedure, including that of methanol, ethanol, or acetone. These findings confirm the unknown to be of chemical nature: a peptide or protein.

**Figure 3:**
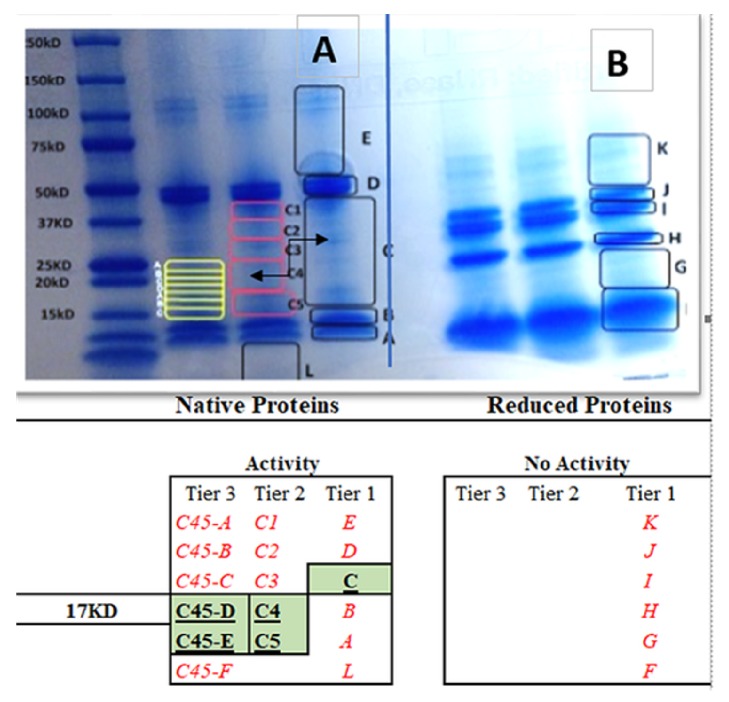
Gel excision layout: total seed proteins (native [A] and reduced) with *β*-Me [B] were separated using a gradient SDS PAGE gel 4–20% Mini-PROTEAN® TGX™ at 200V for 45 minutes. Gels were stained with Blue-band it®, washed in ultrapure water, then excised and electroeluted back into solution at 200V, reconstituted in HBSS, and evaluated for biological neuritogenic activity on PC-12 cells. All gel sections by process of procedural elimination left only two small biologically active (nonvisual) bands at around 16-17kDa (C45D) containing the predominant neuritogenic active fraction.

**Figure 4:**
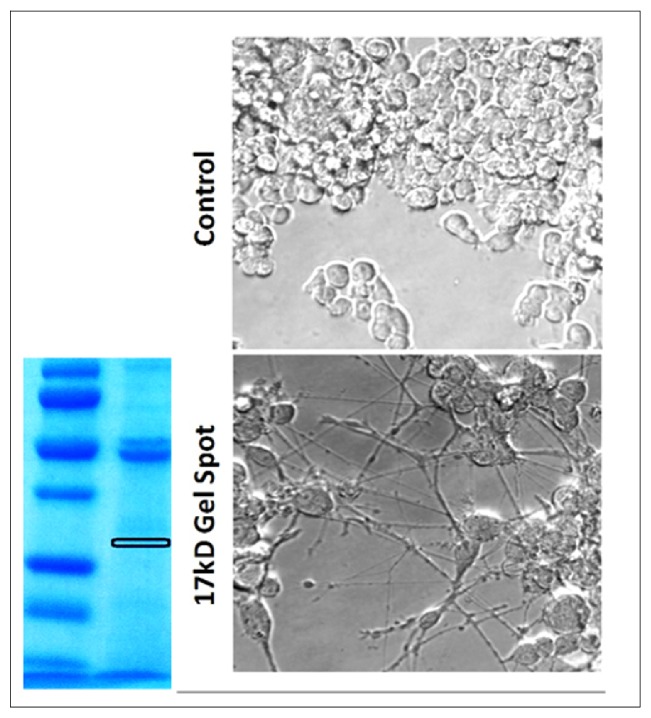
Total seed proteins were separated using a gradient gel 8–16% Mini-PROTEAN® TGX™ gel at 200V for 45 minutes. Gels were stained with Blue-band it®, washed in ultrapure water then excised, and electroeluted back into solution at 200V. Samples were reconstituted in HBSS and evaluated for biological activity on PC-12 cells. The 17kDa band contained the main active protein.

To further separate the crude protein, the entire sample was loaded on to an SDS PAGE gel (native: Figure 3(A); reduced: Figure 3(B)) through a gradient (4-20%), followed by a gel staining procedure (Figure 3(A, B)). Without knowing the stability of the protein, we endeavored to cut the gel into sections, according to the outlines depicted in Figure 4. The first set of gel bands (A-L) Tier 1 were electroeluted back into solution, diluted in HBSS, and evaluated for neuritogenic activity in PC-12 cells. The only fraction of biological activity was B and C (Native gel). In Tier 2 Band c was further cut into five small sections, C1-5, the process was repeated to which neuritogenic activity was found only in bands C4 and C5. In Tier 3, bands C4 and C5 were sectioned into six gel excisions, the process was repeated, and the neuritogenic activity was in gel slice CD45, aligning at 16-17 kD according to the molecular weight protein marker.

Knowing the mass of the unknown protein to be of ~17 kD weight range, separation was repeated and validated using an 8-16% SDS PAGE gel, to where the excised gel band at 17 kD was cut in half (horizontally) revalidated for neurite outgrowth (Figure 4). The other half was analyzed for proteomic content using UPLC-MS/MS on a Q Exactive Hybrid Quadrupole-Orbitrap Mass Spectrometer. The data were searched using X! Tandem, OMSSA, and X Hunter. Analysis of each sequence was also conducted using Basic Local Alignment Search Tool (BLAST) (Table 1). Table 1 shows the confirmed proteins in the 17kDa neuritogenic gel spot. The data represent protein entry ID, description, e-value, intensity, peptides found, identifications, spectral counts, percent coverage, and species/genus of known annotated protein.

## 4. Discussion

The neuroprotective/trophic properties of NGF are well established where the mechanism of action involves it binding to and initiating phosphorylation of tropomyosin-related kinase receptors (Trk) (A-C) and internalization of the receptor complex into lipid raft endosomes, with Rab22GTPase. This process is followed by the activation of pERK (1/2)/cAMP protein kinase A/pCREB signaling which is believed to drive the restructure of microtubule proteins [22–26]. These changes lead to cytoskeletal reorganization/elongation and formation of neuritic shafts embodied by filopodia/lamellipodia growth cones that extend along a biological matrix such as collagen. Subsequently, neurite outgrowth is a gradual process that occurs through repetitive retraction and polymerizing of F-actin aided by hundreds of proteins such as Arp2/3, ccdc8, cortactin, Cap1 and Sept2 Shootin1, GAP-43, fascin, syntaxin 6, or the Rac-cofilin pathway [27–31]. There are likely thousands of events that guide growth cone dynamics and neurite outgrowth, after binding of a growth factor to its cognate receptor.

The endogenous production of neurotrophins such as NGF is essential for repair of central nervous system neurological damage, but its lack of permeability limits the exogenous use of NGF for a therapeutic purpose through the BBB and side effects such as peripheral neuropathies [8]. The search for natural substances or low molecular weight mimetics could lead to new therapies. Recently, in a discovery-based approach, we screened over an 1100 food/plant-based product to determine if any of these could elicit basic neurite outgrowth in PC-12 cells to an extent similar to NGF. We elucidated the seed of* Mu Bie Zi*, Momordica cochinchinensis, while finding no mimetic effects in the fruit [19]. In the current study we explore the biological nature of constituents responsible for neuritogenic effects.

The data in this study corroborate the nature of the neuritogenic factor to be a peptide or protein. This is consistent with previous reports in that neurotrophins are proteins, and mimetics tend to be dimeric peptides or cleaved peptide products [8, 32] suggesting that a protein-protein interaction is imperative to trigger neuritogenic activity. To date, we have not found a single nonprotein, plant-based herb or polyphenolic compound capable of achieving neurite extensions in the PC-12 model.

Several of species under Momordica have reported health benefits with most studies examining the* charantia *fruit which is commonly known as bitter melon, for its ability to antagonize diabetes [33–35], restore glucose homeostasis [36], enhance insulin secretion [37], protect islet beta-cells [38], and aid in healing of diabetic wounds [39]. Bitter melon has additional beneficial health-promoting properties on cardiovascular health [40, 41], gastric ulcers, and can prevent angiogenesis, proliferation, and metastasis in diverse cancers [42–45]. Other species under the Momordica* genus reportedly have *similar health benefits both* M*.* cymbalaria* and* M. dioica* which are also useful in0 reducing pathological aspects of hyperglycemia, diabetes, and gastric ulcers [46–49].

Several Studies of the Momordica cochinchinensis (MC) health benefits of the edible Southeast Asian fruit pulp and red seed cover called the aril were conducted. To date, we know that that aril contains high concentrations of lycopene and beta-carotene [50, 51] and fatty acids (palmitic, oleic, and linoleic) [52, 53]. The peel is high in lutein [53] and the fruit pulp in gallic acid, p-hydroxybenzoic acid, ferulic acid, myricetin, and apigenin. [53] and the root trichostatin and high MW glycoproteins [54, 55]

The seeds contain saponins [56], saponin glycosides [57], and macrocyclic peptides containing disulfide bridges (MCoTI-I and -II trypsin inhibitors) [58, 59]. MCoTI-II are currently under investigation as a drug delivery tool because these small cyclic peptides can enter cells [60] by endocytosis [61] and enter the bloodstream without being cleaved [62]. Drug design transport systems involve grafting a drug onto these cyclic cell-penetrating peptides [60], some being tested for drugs to treat cancer and myocardial infarction [63]. MCoTI-Is by their merit also contains medicinal value as potent anticancer matriptase inhibitors [64] and antimicrobial properties [65].

The seeds also contain nonprotein molecules including fatty acids [52], phytochemicals, karounidiol, isokarounidiol, 5-dehydrokarounidiol, 7-oxodihydrokarounidiol, beta-sitosterol, stigmast-7-en-3beta-ol, stigmast-7,22-dien-3beta-ol [66] lupeol, 5-(1'-hydroxypentyl)-5H-furan-2-one, viscumamide, clavatustide C, laxanol, threo-1-(4-hydroxyphenyl)-2-[67]-propane-1, 3-diol, alpha-spinasterol-3-O-beta-D-glucoside, chushizisin F, ehletianol C, tanegool, (7R, 8R, 8'R)-4'-guaiacylglyceryl-evofolin B, ligballinone, (7R, 8S, 8'R)- 4, 4', 9-trihydroxy- 7, 9'-epoxy- 8, 8'-lignan, chushizisin I, chushizisin A, chushizisin G, p-coumaraldehyde, alpha-spinasterol, p-hydroxybenzoic acid, chushizisin E and 3-[2-(4-hydroxyphenyl)-3-hydroxyphenyl-2, 3-[dihydro-1-benzofuran-5-yl] propane-1-ol [67].

The findings in this study corroborate our previous finding in that the seed also contains a neuritogenic factor [19], further classified as a protein or peptide having a mass of 17 kDa. Given the lack of annotation for the* Momordica cochinchinensis* proteome, identification of proteins was limited to that previously sequenced confirming some sequences similar to the highly ubiquitous 11S globulin and napin seed storage proteins, oleosin, and specific* Momordica genus cyclotides:* trypsin inhibitors I (MCoTI-I), 2 (MCoTI-II), and TIPTOP proteins. The identified proteins mostly fall into a class of defense plant proteins (cyclic cystine-knot proteins) which ensure survival against natural elements, having exceptional thermal stability and resistance to proteolytic degradation, due to the rigid core formed by three disulfide bonds configured in a cystine knot. While trypsin inhibitor characteristics of* Momordica* proteins make them suitable for other medicinal purposes, it is unlikely that the trypsin inhibitor properties of these proteins have anything to do with neurite outgrowth. To examine this, we evaluated (data not shown) for neuritogenic effects of a well-known bovine pancreatic trypsin inhibitor, aprotinin, which failed biological efficacy. Also, due to the lack of a crystal structure for the rat NGF TrKA receptor, docking analysis of elucidated peptides could not be performed.

## 5. Conclusion

In conclusion, the findings obtained demonstrate that the neuritogenic activity inherent to MCS is a protein of 17kDa. Future work will be required to investigate the potential of* Momordica* seed proteins to mitigate pathological processes associated with age-related neurodegenerative diseases.

## Figures and Tables

**Table 1:** Proteomic analysis of the 17 KD spot. The gel was digested, separated, and evaluated by UPLC-MS/MS using and Q Exactive™ Hybrid Quadrupole-Orbitrap Mass Spec. Data were searched using several tandem mass spectrometry protein identification algorithms, including X Tandem and OMSSA/K-score. The data represented protein entry ID, description, E-value, intensity, peptides found, identifications, spectral counts, percent coverage and species, and genus of protein.

**Protein**	**Description**	**E-Value**	**Intensity**	**Peptides**	**ID**	**Spectral Counts**	**Coverage (%)**	**Species Genus**
P13744	11s globulin subunit beta	-38.9	8.53	3	17	12	6.67	Cucumis Sativus
A0A0A0LCF7	Oleosin	-14.7	8.48	1	8	8	6.96	Cucumis Sativus
Q8L694	Napin	-37.2	8.86	3	8	7	17.86	Momordica charantia
P82410	Two inhibitor peptide topologies 5	-21.7	8.79	2	4	34	12.72	Momordica macrophylla
AOAOA7HIA5	Two inhibitor peptide topologies 6	-22.3	8.72	2	5	4	14.16	Momordica macrophylla
P82408	Trypsin inhibitor 1 MCoTI-1	-22.3	8.72	2	5	4	35.29	Momordica cochinchinensis
P82409	Trypsin inhibitor 2 MCOTI-11 Chain A	-22.3	8.72	2	5	4	35.29	Momordica cochinchinensis
J3RCD6	Two inhibitor peptide topologies 1	-22.3	8.72	2	5	4	17.08	Momordica cochinchinensis
J7IN40	Two inhibitor peptide topologies 2	-22.3	8.72	2	5	4	17.88	Momordica cochinchinensis
J3R9Z5	Two inhibitor peptide topologies 3	-27.3	8.72	3	6	4	16.71	Momordica cochinchinensis
AOAOAOL2N7	Non-specific serine/threonine protein kinase	-4.2	6.49	1	1	1	2.4	Cucumis Sativus
AOAOAOKNN9	Elongation factor-1 alpha	-2.3	6.7	1	1	1	2.74	Cucumis Sativus

## Data Availability

The data, in free formats, used to support the findings of this study are available from the corresponding author upon request.

## Abbreviations

AD: Alzheimer's disease
BBB: blood-brain barrier
BDNF: Brain-derived neurotrophic factor
BLAST: Basic Local Alignment Search Tool
CCD: Charge-coupled device
DMEM: Dulbecco's Modified Eagle Medium
FBS: Fetal bovine serum
HBSS: Hanks Balanced Salt Solution
HEPES: 4-(2-Hydroxyethyl)-1-piperazineethanesulfonic acid
HTP: High throughput screening
kDa: Kilodalton
MCS: * Momordica cochinchinensis* seed
NGF: Nerve growth factor
pCREB: cAMP response element binding
PD: Parkinson's disease
SDS PAGE: Sodium dodecyl sulfate-polyacrylamide gel electrophoresis
STAGE: Stop-and-go extraction
TIC: Total ion current
Trk: Tropomyosin-related kinase receptor
UPLC-MS/MS: Liquid chromatography-mass spectrometry.
